# Management of acral lentiginous melanoma: current updates and future directions

**DOI:** 10.3389/fonc.2024.1323933

**Published:** 2024-02-08

**Authors:** Michelle M. Dugan, Matthew C. Perez, Lilit Karapetyan, Jonathan S. Zager

**Affiliations:** ^1^ Department of Cutaneous Oncology, Moffitt Cancer Center, Tampa, FL, United States; ^2^ Department of Oncologic Sciences, University of South Florida Morsani College of Medicine, Tampa, FL, United States

**Keywords:** melanoma, acral melanoma, acral melanoma management, immunotherapy, targeted therapy, regional therapy

## Abstract

Acral lentiginous melanoma is a rare subtype of melanoma generally associated with poor outcomes, even when diagnosed at an early stage. The tumor genetic profile remains poorly understood, but it is known to have a suppressed immune environment compared to that of non-acral cutaneous melanomas, which limits therapy options. There is significant attention on the development of novel therapeutic approaches, although studies are limited due to disease rarity. For local disease, wide local excision remains the standard of care. Due to frequent under-staging on preoperative biopsy, wider margins and routine sentinel lymph node biopsy may be considered if morbidity would not be increased. For advanced disease, anti-PD1 monotherapy or combination therapy with anti-PD1 and anti-CTLA4 agents have been used as first-line treatment modalities. Anti-PD1 and anti-CTLA4 combination therapies have been shown to be particularly beneficial for patients with BRAF-mutant acral lentiginous melanoma. Other systemic combination regimens and targeted therapy options may be considered, although large studies with consistent results are lacking. Regional and intralesional therapies have shown promise for cutaneous melanomas, but studies generally have not reported results for specific histologic subtypes, especially for acral melanoma. Overall, the unique histologic and genetic characteristics of acral lentiginous melanoma make therapy options significantly more challenging. Furthermore, studies are limited, and data reporting has been inconsistent. However, more prospective studies are emerging, and alternative therapy pathways specific to acral lentiginous melanoma are being investigated. As further evidence is discovered, reliable treatment guidelines may be developed.

## 1 Introduction

Acral lentiginous melanoma (ALM) is the rarest of the four major subtypes of cutaneous melanoma, accounting for 2-3% of all melanomas (1–3). ALM occurs predominantly in non-hair-bearing skin of the distal extremities, such as the palms of the hands, soles of the feet, and nailbeds (1, 3–6). This unique histologic subtype was first described by RJ Reed in 1976, as pigmented lesions with a radial (lentiginous) growth phase of melanocytes, which evolves into a dermal (vertical) invasive stage (4, 5, 7).

In addition to its distinctive growth pattern, ALM has additional characteristics separating it from non-ALM cutaneous melanoma. ALM has a much lower mutational burden than non-ALM cutaneous melanomas, including a lower incidence of activating mutations in BRAF and NRAS, variable KIT mutations, and a lack of ultraviolet (UV)-related mutational signatures (3, 7–9). A study by Li et al., utilizing single-cell RNA-sequencing to map the transcriptional landscape of ALM, found a lower overall immune infiltrate, fewer effector CD8 T cells and NK cells, and a near-complete absence of γδ T cells, compared to non-ALM cutaneous melanoma. This study also discovered that ALM and non-ALM cutaneous melanoma cells have different patterns, and overall reduced density, of cell-to-cell communications (8).

Due to disease rarity, poorly understood carcinogenic and immunogenic processes, and underrepresentation in the literature, there is a paucity of level 1 evidence-based guidance (evidence from a systematic review(s) of homogeneous randomized control trials) for managing ALM. Contemporary therapeutic techniques have been generally extrapolated from large, prospective randomized controlled trials on non-ALM cutaneous melanomas, and/or from small retrospective studies on ALM.

### 1.1 Epidemiology

ALM typically presents among patients at an older age, with a mean age at diagnosis of approximately 63 years for ALM compared to approximately 59 years for non-ALM cutaneous melanoma, according to large population-based studies (1, 2, 6, 10). There is a slight female predominance in ALM, and the majority of tumors are found on skin of the lower extremities (1, 6, 7, 10). Pathogenic mutations are unrelated to sun exposure and, consequently, ALM is the most frequent melanoma subtype found in individuals of Asian, African, and Hispanic or Latino descent, with the highest proportion of cases found among those of African descent (1, 3, 7, 9, 11). Geographic distribution is poorly understood due to the scarcity of reported cases, but it is overall reflective of the ethnic origin of the population in that region. For example, studies based in Asia found that ALM represented 55-58% of melanomas in Taiwan and Korea, compared to a population study in the United States which found that ALM represented approximately 2% of melanomas (2, 12, 13). Population-based studies within the United States have found no statistically significant differences in ALM distribution based solely on geographic location (2).

ALM has a significantly lower mutation rate compared to other cutaneous melanomas. Overall, studies worldwide have reported ALM mutation rates in BRAF of 4% to 34%, KIT of 11% to 36%, RAS of 32%, NRAS of 22%, NF1 of 17%, GNAQ of 17% (13–17). The mutation profile of ALM varies between ethnicities as well. Studies based on Asian populations have found lower KIT mutation rates (11% to 12%) compared to those from the United States (12% to 36%) (13–17).

Interestingly, pre-existing benign melanocytic acral nevi are not a risk factor for development of ALM (18). Benign acral nevi have been found to have a significantly higher BRAF mutation rate than in ALM, suggesting that they are not precursor lesions (19). Mechanical stress such as pressure and trauma may play a role in the development of advanced ALM, especially in the lower extremities, but studies have reported conflicting evidence of this potential association (4, 20–22).

### 1.2 Clinical presentation and diagnosis

ALM typically presents among older patients as an asymmetric pigmented lesion on the palms, soles, or nailbeds (4). These lesions have a higher tendency than non-ALM cutaneous melanoma to be ulcerated (7).

Diagnosing ALM can be clinically challenging, as ALM can mimic benign conditions such as ulcers related to vascular disease, diabetes, mechanical pressure, warts, or trauma. Furthermore, since ALM is more common in patients with dark skin pigmentation, lesions may not appear as visually prominent as they would be in patients with light skin tones. Dermoscopy serves as a useful adjunct to differentiate ALM from benign acral nevi, as palmoplantar ALM demonstrates a characteristic parallel ridge pattern and irregular diffuse pigmentation (18, 23). Characteristic dermoscopy findings for subungual ALM include longitudinal brown/black lines, irregular in their coloration, spacing, thickness, and parallelism (24). In the setting of a suspicious pigmented lesion on an acral site that does not respond to a short course of treatment for a suspected benign condition, a prompt biopsy should be undertaken (4).

An excisional biopsy, typically the gold standard for diagnosing melanocytic lesions, can be technically challenging in ALM, since lesion locations typically include sites with a restricted skin reservoir which would require amputation for complete removal of the lesion (4, 18). Therefore, initial biopsy technique is more commonly a punch, shave, or incisional biopsy (18, 25). However, due to the inability to completely evaluate the lesion pathologically, studies have found that a high proportion of patients with ALM had their lesions under-staged on biopsy (18, 26).

Once a biopsy specimen is obtained, histologic findings suggestive of ALM include acanthosis, spindle-cell makeup of the dermal component, poor circumscription, tendency of melanocytes to proliferate singly or in nests, lentiginous growth pattern into the upper epidermis, and inflammatory changes in the papillary dermis (7, 18). Pathologic characteristics must be incorporated into the clinical setting to aid in determining an accurate diagnosis.

For many reasons, often including misdiagnosis for benign conditions, patients with ALM tend to have a delay in diagnosis and are found to have a significantly higher disease stage at diagnosis than patients with non-ALM cutaneous melanoma (1, 7, 10, 18). To overcome these diagnostic challenges, it is critical to have a high level of suspicion for ALM, and to integrate the clinical, dermoscopic, histopathologic, and molecular findings.

### 1.3 Terminology

It is important to note that the term “acral lentiginous melanoma” is not equivalent to “acral melanoma”. The term “acral” refers to a lesion at a peripheral site, such as on the distal extremities. The “lentiginous” designation is reserved for cases with the characteristic radial growth pattern, as described by RJ Reed, and histologic characteristics (1, 4, 5, 7). Patients may have a lesion on the distal extremity appropriately classified as an “acral melanoma”, but without distinguishing features to classify as “lentiginous”, especially if the area has had high levels of ultraviolet and sun exposure. This distinction is frequently not made in the literature, with less than 40% of reports specifying “acral lentiginous melanoma” as a histologic subtype as well as the corresponding appropriate acral anatomic site (27). All efforts were made to focus this review specifically on ALM, with the understanding that some of the literature reviewed may have included non-ALM cutaneous melanoma on acral sites (4, 27).

### 1.4 Clinical outcomes

Large contemporary population-based outcome studies are limited for ALM, with most studies capturing time periods before 2016 (1, 2, 6, 7, 10). Based on currently available literature, ALM is associated with a worse prognosis than non-ALM cutaneous melanoma (1, 2, 7, 10, 11). This may be partially attributed to the tendency of ALM to be associated with a delayed diagnosis, older age, deeper thickness, more frequent ulceration, lymphovascular invasion, lymph node positivity, and higher stage at presentation (1, 6, 7, 28–30). Studies have shown approximately 64% to 68% of non-ALM cutaneous melanoma presenting at stage I, compared to 38% to 45% of ALM presenting at stage I (1, 7). Some studies suggest that ALM has a worse prognosis even despite controlling for variables such as stage and tumor thickness (1, 2, 7, 28). One study found race as a significant prognostic indicator, with significantly lower survival rates among Black patients, after controlling for stage; these patients were found to be older, predominantly men, and with thicker, more ulcerated disease (2). Other prognostic indicators identified include older age, male sex, positive lymph node status, pathologic stage, tumor thickness, ulceration, and socioeconomic status (2, 6, 7, 29, 31).

Population-based studies utilizing the Surveillance, Epidemiology, and End Results (SEER) Program of the National Cancer Institute database found 5-year melanoma-specific survival rates for ALM of approximately 80% to 81%, compared to 91% to 93% for non-ALM cutaneous melanoma (1, 2). Ten-year melanoma-specific survival rates were 67.5% for ALM, compared to 87.5% for non-ALM cutaneous melanoma (1). Slightly different results were found in a population-based study using the National Cancer Database, which found an unadjusted 5-year overall survival (OS) rate of 67.3% for ALM and 75.8% for non-ALM cutaneous melanoma (P<.001) (7). With further stratification by stage, 5-year OS in ALM *vs* non-ALM cutaneous melanoma was 84.6% *vs* 88.6% for stage I (*P<.*001); 62.1% *vs* 64% for stage II (*P=.*7); 47.5% *vs* 56.7% for stage III (*P<.*001); and 16.2% *vs* 16.4% for stage IV (*P=.*02) (7).

## 2 Management

Given the rarity of the disease and inconsistent reporting of ALM in the literature, management techniques have been generally extrapolated from studies on non-ALM cutaneous melanoma, and from fairly small, retrospective studies on ALM. This can be problematic given the inherent distinctions between ALM and non-ALM cutaneous melanoma, including different mutation profiles and tumor microenvironments (3, 7–9). Further research and prospective randomized trials are needed to develop reliable treatment guidelines specific to ALM.

### 2.1 Primary lesion

For localized, resectable disease, upfront surgical resection is standard of care. Margin guidelines have been generally extrapolated from those established for non-ALM cutaneous melanoma. However, given the high frequency of preoperative biopsy under-staging ALM lesions, wider margins and sentinel lymph node biopsy may be considered in patients with lower stage ALM but with residual pigmentation on exam (26). Reflectance confocal microscopy can be useful in the setting of positive margins, as it assists with targeted resection of the area of concern (32).

For subungual ALM, surgical resection with adequate margins typically involves amputation. However, universal amputation has been challenged in recent years. Nakamura et al. found that wide local excision with 0.5 cm to 1 cm peripheral margins and deep margins to underlying bone provided acceptable local control for *in situ* and intermediate thickness invasive disease. Four out of 50 patients with *in situ* disease experienced local recurrence at the lateral margin requiring re-excision, and one out of 12 patients with invasive disease experienced nodal metastasis over 7 years later requiring regional lymph node dissection. No patients with invasive disease experienced local recurrence, no patients required amputation, and all patients survived the follow-up period (24-207 months) (33). A systematic review with meta-analysis comparing Mohs micrographic surgery versus nail unit excision versus amputation for melanoma *in situ* of the nail apparatus found a local recurrence rate of 8.7% (2 of 23 patients) with Mohs micrographic surgery, 4.7% (12 of 254 patients) with nail unit excision, and 2.9% (1 of 34 patients) with amputation. There was no statistically significant difference in local recurrence rates between modalities (34). However, a difference of nearly 6% is likely to be clinically relevant, and the lack of statistical significance is likely related to the small sample sizes. Overall, definitive conclusions cannot yet be drawn based on currently available literature which is significantly limited in study design and power. Further research, including randomized controlled trials, in optimal excision technique for subungual ALM is warranted.

### 2.2 Adjuvant and neoadjuvant therapy

There is limited data regarding the role of systemic therapy in the adjuvant and/or neoadjuvant setting specifically for ALM. While these therapies have been studied extensively for cutaneous melanoma, ALM has served as only a small minority of those patients.

#### 2.2.1 Adjuvant therapy

For stage IIB and IIC cutaneous melanoma, adjuvant therapy with pembrolizumab has been supported by the KEYNOTE-716 trial, and adjuvant therapy with nivolumab has been supported by the CheckMate 76K trial (35, 36). The KEYNOTE-716 trial found that patients with completely resected stage IIB or IIC cutaneous melanoma treated with adjuvant pembrolizumab had a significantly reduced risk of disease progression and death compared to placebo. This study did not report histologic subtype analysis (36). The CheckMate 76K trial treated patients with completely resected stage IIB or IIC cutaneous melanoma with adjuvant nivolumab or placebo. Patients treated with nivolumab had a 58% lower risk of recurrence or death compared to placebo. This study reported 43 patients with ALM, representing 5.4% of the total patient population (35).

For stage III-IV cutaneous melanoma, studies have also supported adjuvant pembrolizumab and adjuvant nivolumab. The EORTC 1325/KEYNOTE-054 phase III trial found a significantly improved recurrence-free survival (RFS) with adjuvant pembrolizumab compared to placebo for resected high-risk stage III melanoma, which led to its approval in the USA and Europe (37). The CheckMate 238 phase III trial compared adjuvant therapy with either nivolumab or ipilimumab for patients with resected stage IIIB-C or IV cutaneous melanoma, and found a significantly improved RFS with nivolumab (38). A 5-year follow-up study of the CheckMate 238 trial confirmed a sustained, long-term improvement in RFS with nivolumab compared to ipilimumab (5-year RFS rates of 50% *vs* 39%). Distant metastasis-free survival rates were 58% and 51%, and 5-year OS rates were 76% and 72% with nivolumab and ipilimumab, respectively (39). None of these studies reported histologic subtype analysis to determine the proportion of ALM within the study population.

The CheckMate 915 trial reaffirmed use of adjuvant nivolumab for resected stage IIIB-D or stage IV cutaneous melanoma. This study found that adjuvant nivolumab monotherapy was superior to combination therapy with ipilimumab (no difference in RFS, and higher rate of treatment adverse events in the combination group). Patients with ALM represented 2.9% of this total study population (n = 54) (40).

A small study by Maeda et al. evaluated 27 patients with ALM treated in the adjuvant setting; 5 patients were treated with nivolumab and 22 patients were treated with either chemotherapy (n = 4), interferon beta (n = 12), or observation (n = 6). This study found no difference in disease-free survival between the groups, although the small sample size limits any clinically significant conclusions (41).

#### 2.2.2 Neoadjuvant therapy

The SWOG S1801 phase II trial studied patients with resectable stage IIIB-IVC cutaneous melanoma, with ALM representing 6% of the total study population; 154 patients were treated with neoadjuvant and adjuvant pembrolizumab, and 159 patients were treated with adjuvant-only pembrolizumab. This study found an event-free survival benefit in patients who received both neoadjuvant and adjuvant pembrolizumab (42).

Neoadjuvant therapy allows for monitoring of *in-vivo* tumor response to treatment, which is particularly useful for ALM, since treatment response is less certain. A large, pooled analysis from the International Neoadjuvant Melanoma Consortium found a significant correlation between a pathologic complete response to neoadjuvant therapy and improved RFS (89% *vs* 50% at 2 years) and OS (95% *vs* 83% at 2 years). In this study, neoadjuvant therapies included ipilimumab and nivolumab combination therapy (n = 104), anti-PD1 monotherapy (n = 37), and targeted therapy (n = 51). The pathologic complete response rates were 47% for targeted therapy, 43% with combination therapy, and 20% with anti-PD1 monotherapy. Histological subtype analysis was not reported in this study (43). This concept was further emphasized in a study by Huang et al., where patients with stage III/IV cutaneous melanoma who developed a “response signature”, a specific immune response measured on a blood sample at three weeks after one dose of neoadjuvant anti-PD1 therapy, had a lower disease recurrence than those who displayed mechanisms of resistance. This study did not report histologic subtype analysis (44).

The OpACIN-neo phase II trial studied three different dosing schedules for ipilimumab and nivolumab combination neoadjuvant therapy for patients with stage III melanoma. The radiological objective response rates ranged from 35-65% and the pathological response rates ranged from 65-80%, depending on the dosing schedule. The highest radiological and pathological response rates were seen in group A, with a regimen of two cycles of ipilimumab (3 mg/kg) plus nivolumab (1 mg/kg) once every three weeks intravenously. This study did not report histologic subtype analysis (45).

The PRADO trial, an extension cohort of the OpACIN-neo trial, 99 patients with stage IIIB-D nodal cutaneous melanoma were treated with neoadjuvant ipilimumab and nivolumab. The pathologic response rate was 72%, and the major pathologic response (¾ 10% viable tumor in their index lymph node) rate was 61%. Patients with a major pathologic response underwent no additional therapy. Patients with a pathologic partial response (10-50% viable tumor) underwent therapeutic lymph node dissection. Patients with pathologic non-response (>50% viable tumor) underwent therapeutic lymph node dissection, adjuvant systemic therapy, and possibly synchronous radiotherapy. Based on two-year relapse-free survival rates and distant metastasis-free survival rates, patients with major pathologic response could safely omit therapeutic lymph node dissection and adjuvant therapy. This study did not report histologic subtype analysis (46).

A study by Amaria et al. analyzed a combination therapy regimen with relatlimab (a LAG-3 inhibitor) and nivolumab in the neoadjuvant setting for patients with resectable stage III or oligometastatic stage IV cutaneous melanoma. This study found a pathologic complete response rate of 57% and an overall pathologic response rate of 70%. This study did not report histologic subtype analysis (47).

Ultimately, this area of interest requires more data from ALM-focused research trials to more fully evaluate the role of systemic therapy in the adjuvant and/or neoadjuvant setting.

### 2.3 Advanced disease

For unresectable, locally advanced, or metastatic ALM, systemic therapy options should be considered. Unfortunately, the immune microenvironment in ALM remains poorly understood, and studies have shown lower response rates and shorter response durations with ALM to systemic therapy, compared to non-ALM cutaneous melanoma (8, 48).

### 2.4 Immune checkpoint inhibitor therapy

#### 2.4.1 Anti-CTLA4 monotherapy

Studies have found that anti-CTLA4 agents, such as ipilimumab, used as monotherapy are not as effective as anti-PD1 monotherapy or a combination of anti-PD1 and anti-CTLA4 therapy (**Table 1**). In a large Australian meta-analysis by Cho et al., evaluating 646 patients with ALM, patients who underwent anti-PD1 monotherapy had a significantly higher OS at 1-year compared to those who underwent anti-CTLA4 monotherapy (53% *vs* 34%, P<.001) (48). A smaller study by Wen et al., which studied 22 patients with ALM, treated 7 patients with ipilimumab and 13 patients with pembrolizumab. The ORR among patients treated with ipilimumab was 0%, compared to an ORR of 26.7% for patients treated with pembrolizumab (56). A multi-institutional study by Bhave et al. analyzed 325 patients with ALM who received either ipilimumab monotherapy (n = 82), anti-PD1 monotherapy (n = 184), or ipilimumab/anti-PD1 combination therapy (n = 59). Patients who received ipilimumab monotherapy had a significantly lower ORR of 15% compared to the other cohorts (26% for anti-PD1 and 43% for combination therapy) and lower PFS at 1-year of 10% and 2-years of 6% (compared to 1-year and 2-year PFS of 26% and 18% for anti-PD1, and 34% and 22% for combination therapy) (61). A study by Yamazaki et al. treated 107 patients with ALM with ipilimumab monotherapy, and found a notably low median OS of 7.2 months (51).

**Table 1:** Key studies for advanced acral lentiginous melanoma.

Author	Region, year	Study design	Sample size (ALM)	Therapeutic agent(s)	Line of study therapy (median)	Prior ipilimumab	ORR	Median PFS (months)	Median OS (months)	Median follow-up (months)	Other
Immune Checkpoint Inhibitor Therapy											
Si et al. (49)	China 2022	Prospective (3-year follow up of KEYNOTE- 151)	38	Pembrolizumab PDL1(+) (n = 19) PDL1(-) (n = 16) BRAF-wild (n = 34) BRAF-mutant (n = 4)	Second	17%^a^	Overall: 18.4% PDL1(+): 26.3% PDL1(-):12.5% BRAF-wild: 20.6% BRAF-mutant: 0%	Overall: 2.8 PDL1(+): 4.4 PDL1(-): 2.7 BRAF-wild: 3.4 BRAF-mutant: 1.9	Overall: 14.8 PDL1(+): 22.8 PDL1(-): 8.4 BRAF-wild: 18.5 BRAF-mutant: 5.8	44.6	DCR Overall: 42.1% DCR for PDL1(+): 52.6% DCR for PDL1(-): 31.3% DCR for BRAF-wild: 47.1% DCR for BRAF-mutant: 0%
Cho et al. (48)	Australia 2021	Meta-analysis, retrospective	646	Nivolumab or pembrolizumab vs anti-CTLA4	N/A	N/A	N/A	N/A	15	N/A	OS at 1-year: 53% vs 34%
Nakamura et al. (50)	Japan 2020	Retrospective	193	Nivolumab or pembrolizumab	First	4%	16.6%	3.5	18.1	11.4	
Yamazaki et al. (51)	Japan 2020	Retrospective	107	Ipilimumab	N/A	N/A	N/A	N/A	7.2	N/A	
Tang et al. (52)	China 2020	Prospective phase II (POLARIS-01)	50	Toripalimab	Third	7%^a^	14%	3.2	17	>23^a^	
Si et al. (53)	China 2019	Prospective phase Ib (KEYNOTE-151)	38	Pembrolizumab	Second	17%^a^	15.8%	N/A	N/A	7.9	
Nathan et al. (54)	Europe 2019	Prospective phase II (CheckMate 172)	55	Nivolumab	Third	100%	N/A	N/A	25.8	18.5	
Maeda et al. (55)	Japan 2019	Retrospective	16	Nivolumab	N/A	N/A	19%	6.6	14	N/A	
Wen et al. (56)	China 2017	Retrospective	22	Ipilimumab (n = 7) vs pembrolizumab (n = 13) vs combination (n = 2)	N/A^b^	N/A^b^	Ipilimumab: 0% Pembrolizumab: 26.7% Combination: N/A	Overall: 3	Not reached	11^a^	
Shoushtari et al. (57)	USA 2016	Retrospective	25	Nivolumab or pembrolizumab	Third	77%	32%	4.1	32	20	
Combination Therapy											
Mao et al. (58)	China 2023	Prospective phase II (CAP 03 trial)	50	Camrelizumab, apatinib, and temozolomide	First	0%	64%	18.4	Not reached	13.4	DCR 88% Median time to response: 2.7 months Median DOR: 17.5 months
Wang et al. (59)	China 2023	Prospective phase II	30	Apatinib and camrelizumab	First	0%	24.1%	7.4	13.4	26.1	DCR 82.8%
Nakamura et al. (60)	Japan 2022	Retrospective	254	Anti-PD1 (n = 209) vs anti-PD1 + anti-CTLA4 (n = 45) Palms/Soles (n = 180) Nail apparatus (n = 74)	First	N/A	Overall: 16% vs 40% Palms/soles: 19% vs 31% Nail apparatus: 10% vs 61%	Overall: 4.7 vs 6.6 Palms/soles: 5.9 vs 3.2 Nail apparatus: 3.8 vs 8.4	Overall: 20.7 vs 43.6 Palms/soles: 23.1 vs not reached Nail apparatus: 13.2 vs 23.1	Anti-PD1: 13.1 Anti-PD1 + anti-CTLA4: 11.3	Palms/soles OS at 2 and 3 years: 45% vs 63% (2-years) and 28% vs 63% (3-years)
Bhave et al. (61)	International (USA, Australia, China, Europe) 2022	Retrospective	325	Ipilimumab (n = 82) vs anti-PD1 (n = 184) vs combination (n = 59)	First	N/A	Ipilimumab: 15% Anti-PD1: 26% Combination: 43% BRAF-mutant (n = 38): Ipilimumab (n = 7): 67% Anti-PD1 (n = 10): 31% Combination (n = 21): 63%	Overall: 4 Ipilimumab: 3.5 Anti-PD1: 4.1 Combination: 5.4 BRAF-mutant: 5.1	Overall: 22.8 Ipilimumab: 22.8 Anti-PD1: 22.8 Combination: 15.6 BRAF-mutant: 54	46.8	PFS at 1, 2, and 5 years Overall: 23.3% (1-year), 6.4% (5-years) Ipilimumab: 10%/6%/NA Anti-PD1: 26%/18%/7% Combination: 34%/22%/18% OS at 1, 2, and 5 years Overall: 68.2% (1-year), 22.5% (5-years) Ipilimumab: 68%/48%/21% Anti-PD1: 69%/49%/28% Combination: 66%/43%/16%
Tawbi et al. (62)	International (USA, Europe, Australia, South/Central America) 2022	Prospective phase II-III	82	Relatlimab + nivolumab (n = 41) vs nivolumab monotherapy (n = 41)	First	N/A	N/A	N/A	N/A	N/A	PFS benefit in combination therapy group
Targeted Therapy											
Mao et al. (63)	China 2021	Prospective (follow-up of phase IIa trial)	12 (BRAF V600 mutant)	Dabrafenib and trametinib	Second^a^	N/A	35.7%	At 2 years, 10 out of 12 progressed	At 3 years, 35.7%	37^a^	
Bai et al. (64)	China 2017	Retrospective	28 (BRAF V600Emutant)	BRAF inhibitor (vemurafenib, sorafenib, or BGB-283)	First	N/A	38.1%	3.6	6.2	N/A	DCR 81%
Kim et al. (65)	South Korea 2016	Retrospective	10 (BRAFV600Emutant)	Dabrafenib and trametinib (n = 11), or vemurafenib (n = 16)	First	N/A	78.9%^c^	7.3^c^	N/A	32.1^a^	Median DOR: 4.5 months PFS 50.2% at 6 months^c^
Steeb et al (66)	Germany 2021	Meta-analysis, retrospective	109	Imatinib (n = 80), nilotinib (n = 113), or dasatinib (n = 48)	N/A	N/A	22%	2.8	21.1	N/A	

ALM, acral lentiginous melanoma; DOR, duration of response; NA, not applicable; ORR, overall response rate; OS, overall survival; PFS, progression-free survival.

a Value reported for overall patient population with all melanoma subtypes, not specific to ALM.

b Based on pooled patient population, >50% in each therapy line had received prior therapy with chemotherapy or BRAF inhibitors; not specific to ALM cohort.

c Value reported for patients with acral and mucosal melanomas combined.

#### 2.4.2 Anti-PD1 monotherapy

Use of anti-PD1 therapeutic agents, such as pembrolizumab and nivolumab, for ALM was first adopted due to landmark trials mostly focused on non-ALM cutaneous melanoma. Studies have shown that ALM has a lower frequency of PD-L1 expression compared to non-ALM cutaneous melanoma (33% *vs* 62%) (67). Despite this, subsequent trials specific to ALM patients have shown promising results with anti-PD1 agents (**Table 1**).

In general, ORR, median PFS, and median OS with anti-PD1 therapy have been found to be lower in studies from China and Japan compared to those from Europe and the United States. Studies from China and Japan using nivolumab or pembrolizumab for ALM have found an ORR generally between 14% to 19% (one small study with 26.7%), median PFS between 2.8 and 6.6 months, and median OS between 14 and 18.1 months (49, 50, 52, 53, 55, 56). This is compared to an ORR of 32% and median PFS of 4.1 months from a United States-based study, and a median OS of 25.8 to 32 months from United States and European-based studies (54, 57).

The KEYNOTE-151 trial, a phase 1b study in China evaluating pembrolizumab as second-line therapy for advanced melanoma, with ALM representing 37.9% of their total study population, found an ORR of 15.8% for ALM (n = 38) (53). In a three-year follow-up study, the ORR for ALM was 18.4% (n = 38), median PFS was 2.8 months, median OS was 14.8 months, and disease control rate (DCR) was 41.2%. Patients were further stratified by PD-L1 and BRAF status. Patients with ALM and PD-L1-positive disease *vs* PD-L1-negative disease had an ORR of 26.3% *vs* 12.5%, a median PFS of 4.4 months *vs* 2.7 months, median OS of 22.8 months *vs* 8.4 months, and DCR of 52.6% *vs* 31.3%. Patients with ALM and BRAF wild-type disease *vs* BRAF-mutant disease had an ORR of 20.6% *vs* 0%, a median PFS of 3.4 months *vs* 1.9 months, median OS of 18.5 months *vs* 5.8 months, and DCR of 47.1% *vs* 0% (49).

Similar findings were reported in a Japanese study by Nakamura et al., which retrospectively analyzed 193 patients with ALM who received nivolumab or pembrolizumab as first line therapy, with an ORR of 16.6%, a median PFS of 3.5 months, and median OS of 18.1 months (50). A smaller Japanese study by Maeda et al., which retrospectively studied 16 patients with ALM who received nivolumab therapy, found an ORR of 19%, a median PFS of 6.6 months, and median OS of 14 months (55). A Chinese study by Wen et al. retrospectively studied 13 patients with ALM who underwent therapy with pembrolizumab, with a uniquely high ORR of 26.7% (56). A prospective phase II study in China by Tang et al. analyzed 50 patients with ALM who received a less commonly used anti-PD1 therapy drug, toripalimab, as a later line therapy, with an ORR of 14%, a median PFS of 3.2 months, and median OS of 17 months (52).

The CheckMate 172 trial, a phase II study based in Europe, with ALM representing 5.5% of their total study population, evaluated the use of nivolumab for advanced melanoma with progression on or after ipilimumab treatment. Median OS for ALM was 25.8 months (n = 55) (54). A United States-based multi-institutional retrospective cohort analysis by Shoushtari et al. showed similar findings, in which 25 patients with ALM were treated with nivolumab or pembrolizumab (85% treated with prior therapy, 77% with prior ipilimumab), with an ORR of 32%, a median PFS of 4.1 months, and median OS of 32 months (57).

#### 2.4.3 Combination therapy versus monotherapy

Overall, combination therapy for advanced ALM has shown better efficacy than monotherapy, and is therefore the current standard of care (**Table 1**). Some combinations have included anti-PD1 and anti-CTLA4 agents, but others have incorporated newer mechanisms such as apatinib (a tyrosine kinase inhibitor that selectively inhibits vascular endothelial growth factor receptor-2), temozolomide (DNA-alkylating agent), and relatlimab (a LAG-3 inhibitor).

A large international study by Bhave et al., based on institutions from the United States, Australia, and Europe, retrospectively studied 325 patients with ALM who received either ipilimumab monotherapy, anti-PD1 monotherapy, or ipilimumab/anti-PD1 combination therapy. This study found a significantly higher ORR of 43% for the combination therapy group, *vs* 26% for the anti-PD1 monotherapy group, and 15% for the ipilimumab monotherapy group. PFS followed a similar trend, with a median PFS of 5.4 months, 4.1 months, and 3.5 months for the combination therapy group, anti-PD1 monotherapy group, and ipilimumab monotherapy group, respectively. With combination therapy, PFS was 34% at 1-year, 22% at 3-years, and 18% at 5-years. With anti-PD1 monotherapy, PFS was 26% at 1-year, 18% at 3-years, and 7% at 5-years. With ipilimumab monotherapy, PFS was 10% at 1-year, 6% at 3-years, and not evaluable at 5-years. This trend did not correlate with an OS advantage. Notably, patients with BRAF-mutant disease had better responses to all lines of therapy (ORR of 63% to combination, 31% to anti-PD1, and 67% to ipilimumab therapy *vs* 43% to combination, 26% to anti-PD1, and 15% to ipilimumab for all patients) and longer median OS, 4.5 years *vs* 1.9 years for all patients (61).

A large Japanese study by Nakamura et al. analyzed 254 patients with ALM who received either anti-PD1 monotherapy or anti-PD1/anti-CTLA4 combination therapy. Patients in the combination therapy group had a significantly higher ORR (40%) compared to patients in the monotherapy group (16%), *P=.*01. Patients in the combination therapy group also had a higher median PFS (6.6 *vs* 4.7 months) and median OS (43.6 *vs* 20.7 months), although neither was statistically significant. Of note, this study found that ALM of the nail apparatus responded particularly well to combination therapy compared to monotherapy (ORR 61% *vs* 10%, *P<.*001), although this did not correspond with statistically significant differences in PFS or OS. This ORR difference was not seen among patients with ALM of the palms or soles (31% *vs* 19%, P=.4) (60).

A recent Chinese prospective phase II trial by Mao et al. studied combination therapy with camrelizumab, apatinib, and temozolomide as first-line therapy for 50 patients with ALM. This study found a particularly high ORR of 64%, median PFS of 18.4 months, DCR of 88%, median time to response of 2.7 months, and median duration of response of 17.5 months (58). A similar recent prospective phase II study by Wang et al., also out of China, studied 30 patients with ALM treated with first-line combination therapy with apatinib and camrelizumab. This study found an ORR of 24.1%, a median PFS of 7.4 months, median OS of 13.4 months, and DCR of 82.8% (59).

A prospective phase II/III study by Tawbi et al. treated patients with advanced melanoma with either relatlimab and nivolumab combination therapy or nivolumab monotherapy. This study found a PFS benefit in the combination therapy group for all patients (n = 714) as well as for subgroups such as ALM (n = 82) (62).

### 2.5 Targeted molecular therapy

Like most therapy options used for ALM, targeted therapy models were based on those established for cutaneous melanoma. However, ALM has a lower somatic mutation rate than non-ALM cutaneous melanoma, so these therapies generally play a more limited role (68). Furthermore, ALM harbors heterogeneity in BRAF mutations, which can be distinct from V600E/V600K, commonly found in non-ALM cutaneous melanoma (69). In the relatively infrequent cases of ALM harboring BRAF mutations, the level of response to BRAF and BRAF-MEK inhibition is similar to BRAF-mutant non-ALM cutaneous melanoma, although the length of response tends to be shorter for ALM than non-ALM cutaneous melanoma (8).

In a prospective follow-up of a phase II Chinese study by Mao et al., 12 patients with BRAF-V600-mutant ALM were treated with a combination therapy of dabrafenib and trametinib, with an ORR of 35.7%, a progression of 83% at 2-years, and OS of 35.7% at 3-years (63). Similar results were found in another Chinese study by Bai et al., retrospectively analyzing 28 patients with BRAF-V600E-mutant ALM who were treated with either vemurafenib, sorafenib, or BGB-283. This study found an ORR of 38.1%, a median PFS of 3.6 months, median OS of 6.2 months, and DCR of 81% (64).

A retrospective study by Kim et al. based in South Korea analyzed 10 patients with BRAF-V600E-mutant ALM, treated first-line with either dabrafenib and trametinib or vemurafenib. This study reported results combining acral and mucosal melanomas (n = 19), with an ORR of 78.9%, a median PFS of 7.3 months, PFS at 6 months of 50.2%, and median duration of response of 4.5 months (65).

Genomic alterations in the receptor tyrosine kinase KIT have also been identified in ALM. In a systematic review by Steeb et al., which included studies investigating c-KIT inhibitor targeted therapy agents such as imatinib, nilotinib, and dasatinib, found an ORR of 22% (based on eight studies, n = 109 patients with ALM), a median PFS of 2.8 months (based on one study, n = 21 patients with ALM), and median OS of 21.1 months (based on one study, n = 21 patients with ALM) for patients with ALM. For ALM, imatinib showed a slightly higher ORR (27%) than nilotinib (22%). Objective responses were almost exclusively achieved by patients with KIT mutations in exons 11 and 13 (66).

### 2.6 Regional and intralesional therapy

The nature of ALM and its recurrence patterns in the distal extremities make it particularly amenable to regional therapy approaches (4). However, few studies have evaluated regional therapies specifically for ALM, and data are limited.

#### 2.6.1 Isolated limb infusion

In a large study based in China by Li et al., 150 patients with cutaneous melanoma with in-transit metastases (with ALM representing 79% of the total study population) received an isolated limb infusion (ILI), with a 6% complete response (CR) rate and a 35% partial response (PR) rate. Patients with CR or PR to ILI had better in-field PFS and OS. Stage IV disease and higher burden of disease were associated with worse in-field PFS and OS (70). Compared to this study, where the vast majority of patients had ALM, studies focused on ILI for all cutaneous melanomas with in-transit metastases have found much higher CR and PR rates. Miura et al. studied 687 patients who underwent an ILI for cutaneous melanoma with in-transit metastases, and found a CR rate of 28.9% and a PR rate of 35.2%, with an ORR of 64.1% (71). Carr et al. reported on patients who underwent an ILI for cutaneous melanoma with in-transit metastases in the USA (n = 276) and Australia (n = 411), with a 29% CR, 24% PR, and 53% ORR in the USA, and a 30% CR, 43% PR, and 73% ORR in Australia (72). This indicates that ALM may be less responsive to ILI therapy than non-ALM cutaneous melanomas, although further studies are needed to explore this.

#### 2.6.2 Intralesional talimogene laherparepvec

Intralesional talimogene laherparepvec (T-VEC), an oncolytic virus immunotherapy, has also been studied, both as monotherapy and in combination with systemic therapy. T-VEC was approved by the FDA after the landmark OPTiM trial, out of the United States, which found that T-VEC was well tolerated among 436 patients, and resulted in a higher DRR and longer median OS when compared to GM-CSF (73). However, this study did not account for histologic subtype, to draw conclusions specific to ALM. In The Netherlands, Franke and colleagues reported a case study of a patient with ALM who achieved a histopathologically confirmed complete response to T-VEC as first-line therapy (74).

A multi-institutional phase II study based in the United States and Europe, by Chesney et al., found that combining T-VEC with ipilimumab had a significantly higher ORR than treatment with ipilimumab alone (39% *vs* 18%, P=.002) (75). A more recent phase III trial, however, found that combination T-VEC with pembrolizumab did not significantly improve PFS or OS compared to placebo with pembrolizumab (76). Ultimately, though, neither of these studies reported on histologic subtypes, so it is unclear what proportion of ALM these studies represent.

#### 2.6.3 Radiation therapy

Literature is severely lacking regarding the role of radiation therapy specifically in ALM. One case series from 1999 described four patients with unresectable ALM of the foot, and reports excellent responses to palliative radiation therapy (77). Currently, radiation therapy is most commonly used as adjuvant therapy for recurrent or metastatic melanoma, or for symptom palliation of metastatic disease. Further studies are needed to determine the potential role of upfront radiation therapy in unresectable ALM.

#### 2.6.4 Electrochemotherapy

Electrochemotherapy was designed to increase cell permeability by applying an electrical current to tissues, thereby enhancing cytotoxicity of the locally administered chemotherapy agent. A case series reported two patients with advanced melanoma of the lower extremity, previously non-responsive to immunotherapy and isolated limb perfusion, who had a positive clinical response to bleomycin electrochemotherapy (78). Another case series of 31 patients with unresectable locoregional recurrent or metastatic melanoma, treated with bleomycin electrochemotherapy, found a PR rate of 49%, CR rate of 23%, and disease progression in 28%. ALM represented 13% of this study’s patient population (n = 4), of those, one patient had progression, two had PR, and one had CR (79). Overall, literature specifically related to electrochemotherapy for ALM is currently lacking, and further studies are needed.

## 3 Discussion

In general, ALM seems to respond to anti-PD1 therapy, more specifically with combination therapy regimens, typically including anti-PD1 therapy with anti-CTLA4 therapy but at a lower frequency than when used for cutaneous melanomas (49, 50, 52, 53, 55–57, 60–62). Patients with PD-L1 positive and/or BRAF-wild-type tumors have been found to have particular benefits with anti-PD1 therapy, including higher ORR, longer median PFS, longer median OS, and higher DCR when compared to patients with PD-L1 negative or BRAF-mutant tumors (49).

Results from large, recent studies have found significantly higher ORR with anti-PD1 and anti-CTLA4 combination therapy, compared to monotherapy (40%-43% *vs* 15%-26%), and longer PFS, although this did not consistently result in a median OS benefit (60–62). Of note, one large international study by Bhave et al. found that patients with BRAF-mutant tumors had superior response rates and a far superior median OS (54 months *vs* 22.8 months) for all lines of therapy (ipilimumab monotherapy, anti-PD1 monotherapy, and ipilimumab/anti-PD1 combination therapy) as well as overall results (61). This is in contrast to a smaller study analyzing BRAF-mutant tumors treated with anti-PD1 therapy alone, which found worse outcomes in BRAF-mutant disease (although sample size was prohibitively small for BRAF-mutant disease, with n = 4) (49). Therefore, the combination regimen with anti-CTLA4 agent may be particularly important for patients with BRAF-mutant disease. Bhave et al. found that the ORR for ipilimumab monotherapy was 67% in BRAF-mutant tumors, compared to 15% in the entire patient cohort. Furthermore, the ORR for combination therapy was 63% in BRAF-mutant disease compared to 43% for the entire patient cohort (61).

Other combinations including medications such as camrelizumab, apatinib, and temozolomide have also shown promising results (58, 59). A prospective study treating patients with a combination of camrelizumab, apatinib, and temozolomide found a particularly high ORR of 64% and DCR of 88% (58). A similar DCR was found in a study using a regimen of apatinib and camrelizumab, but the ORR was found to be lower, at 24.1% (59). Overall, studies are currently limited and further evidence is needed to analyze these therapy regimens.

Patients with BRAF V600E-mutant ALM might be good candidates for targeted BRAF inhibitor therapy. The ORR found among studies ranges from 35.7% to 78.9%, with one DCR of 81%, but a median OS of 6.2 months (63–65). These studies have small sample sizes and are mostly retrospective, so further data are necessary to determine the true significance of these findings.

### 3.1 Future directions

New targeted therapies with actionable targets specific to ALM are currently being investigated. Cellular pathways associated with a pathogenic role in ALM include MAPK, PI3K/AKT/PTEN, JAK/STAT3, TERT, WNT, CDK4/CDKN2A, MDM2/TP53, and MCR1-MITF (80). PI3K/AKT/mTOR inhibitors, CDK inhibitors, and MDM/p53 inhibitors, are also being studied (81–83). New immune checkpoint inhibitors could also be adapted to target specific checkpoints found in ALM (other than PD1 and CTLA4). Li et al. used single-cell RNA-sequencing to discover that ALM immune cells expressed additional therapeutically tractable checkpoints, including LAG-3, VISTA, TIGIT, and ADORA2. This study found that VISTA was expressed in 58.3% of myeloid cells, TIGIT was expressed in 22.3% of T/NK cells, and LAG-3 was expressed in 12.9% of T/NK cells (8). Ultimately, however, these may have limited use given the high rate of tumor heterogeneity, with different mutation profiles in various regions even within the same tumor (8, 69, 84).

There are notable differences in response rates to PD1 blockade that could be based on ethnicity and geographic location (85). It is critical to incorporate global inclusivity in future studies to fully evaluate these differences and tailor individualized treatments.

## 4 Conclusion

ALM is a rare melanoma subtype with a traditionally poor prognosis. In general, ALM seems to respond to anti-PD1 therapy, more specifically with combination therapy regimens, typically including anti-PD1 therapy with anti-CTLA4 therapy (49, 50, 52, 53, 55–57, 60–62). Other combinations including medications such as camrelizumab, apatinib, and temozolomide have also showed particularly promising results, but need further analysis (58, 59). Patients with BRAF V600E-mutant ALM might be good candidates for targeted BRAF inhibitor therapy, although more studies are needed to support this (63–65).

Overall, current prospective data for ALM are limited. To gain a deeper knowledge of this disease process and treatment response, it is critical to develop more randomized trials specific to ALM. It is also important that future studies incorporate global inclusivity to fully evaluate potential differences in response rates across different geographic regions and ethnic backgrounds.

## Author contributions

MD: Conceptualization, Methodology, Project administration, Writing – original draft. MP: Conceptualization, Methodology, Project administration, Writing – review & editing. LK: Conceptualization, Methodology, Project administration, Writing – review & editing. JZ: Conceptualization, Methodology, Project administration, Supervision, Writing – review & editing.
